# Effect of distal cantilever and anterior implant diameter on framework and cortical bone stress in a PEEK-based All-on-Four mandibular prosthesis

**DOI:** 10.3389/fdmed.2026.1732749

**Published:** 2026-04-01

**Authors:** Shobha Rodrigues, Sandipan Mukherjee, Reon David Sequeira, Krishna Kumar P, Nithesh Naik, Prashant Bajantri, Sowmya N, Shivani Shetty, Vathsala Patil

**Affiliations:** 1 Department of Prosthodontics, Manipal College of Dental Sciences Mangalore, Manipal Academy of Higher Education, Manipal, India; 2 Manipal Institute of Technology, Manipal Academy of Higher Education, Manipal, India; 3 Department of Oral Medicine and Radiology, Manipal College of Dental Sciences, Manipal Academy of Higher Education, Manipal, India

**Keywords:** All-on-Four, biomechanical performance, cantilever, finite element analysis, implant diameter, mandibular prosthesis, PEEK framework

## Abstract

**Background:**

Polyetheretherketone (PEEK) has been proposed as an alternative framework material for full-arch implant-supported prostheses due to its lower elastic modulus relative to metallic frameworks. However, quantitative data regarding the combined biomechanical influence of distal cantilever extension and anterior implant diameter in PEEK-based mandibular All-on-Four rehabilitations remain limited.

**Purpose:**

To quantify the effects of distal cantilever presence and anterior implant diameter (3.5 mm vs. 4.5 mm) on framework stress and peri-implant cortical bone response in a PEEK-based mandibular All-on-Four prosthesis using three-dimensional finite element analysis.

**Methods:**

Four implant configurations were modeled: Standard-NC (4.5 mm anterior, no cantilever), Mixed-NC (3.5/4.5 mm anterior, no cantilever), Standard-C (4.5 mm anterior, with cantilever), and Mixed-C (3.5/4.5 mm anterior, with cantilever). A 300 N static load was applied at premolar and molar positions under vertical (0°) and 30° oblique loading. Peak von Mises stress in the PEEK framework and crestal cortical bone, along with maximum principal cortical strain, were recorded.

**Results:**

Distal cantilever extension increased peak framework stress by up to 59% under premolar vertical loading (222.24 MPa vs. 139.60 MPa) and increased crestal cortical stress by approximately 49% (18.36 MPa vs. 12.31 MPa) compared with the non-cantilevered baseline configuration. Under molar vertical loading, maximum principal strain increased by approximately 105% in the Mixed-C model compared to the Standard-NC configuration (0.0080 vs. 0.0039). Reduction of anterior implant diameter increased cortical strain by approximately 28–31% in non-cantilevered models and further amplified strain when combined with cantilever extension. Peak framework stresses reached 222.24 MPa in localized regions.

**Conclusion:**

Distal cantilever extension was the primary determinant of biomechanical amplification in a PEEK-based mandibular All-on-Four prosthesis, while reduced anterior implant diameter exerted a secondary but additive effect. These findings highlight the mechanical sensitivity of low-modulus framework systems to cantilever geometry and implant diameter in full-arch rehabilitations.

## 1 Introduction

Edentulism remains a significant global oral health concern, adversely affecting mastication, speech, facial aesthetics, and overall quality of life (1). Implant-supported full-arch prostheses have become a predictable treatment modality, with modern reviews reporting cumulative survival rates exceeding 95%, for completely edentulous patients, providing improved function and patient satisfaction compared with conventional removable dentures (2). Among available protocols, the all-on-four concept, first described by Malo et al. (3), has gained widespread clinical acceptance by enabling full-arch rehabilitation using four implants while minimizing the need for bone augmentation. The technique involves placement of two anterior axial implants and two posterior tilted implants (30°–45°), thereby increasing anteroposterior spread and improving load distribution (4, 5).

Despite favorable clinical outcomes, mechanical complications continue to be reported, particularly in association with distal cantilever extensions and framework biomechanics. Distal cantilevers increase bending moments and stress concentration at the implant abutment interface, which may adversely affect peri-implant cortical bone and compromise long-term prosthetic stability (6–8). Additionally, implant diameter influences load transfer capacity by altering cross-sectional area and structural stiffness. Reduction in anterior implant diameter may be clinically necessary in cases of limited bone availability, yet its interaction with cantilever extension in full-arch rehabilitations remains incompletely quantified.

Consequently, framework material selection plays a critical role in governing load transfer and stress distribution within implant-supported full-arch prostheses.

Metallic frameworks such as titanium, cobalt–chromium, and nickel–chromium alloys (approximately 110–200 GPa) have traditionally been used due to their high strength and rigidity (9, 10). However, their elastic moduli substantially exceed that of cortical bone, which may contribute to stress shielding and unfavorable load transmission (11–14). These biomechanical concerns, along with esthetic limitations and potential metal hypersensitivity, have prompted investigation into alternative materials.

Polyetheretherketone (PEEK), a high-performance thermoplastic belonging to the polyaryletherketone (PAEK) family, has emerged as a promising metal-free framework material in implant prosthodontics (15–22). With an elastic modulus of approximately 3–4 GPa, PEEK more closely approximates that of cortical bone and may facilitate more favorable stress distribution. It also exhibits radiolucency, chemical stability, biocompatibility, and compatibility with computer-aided design and manufacturing workflows (18–20). However, its lower stiffness relative to metallic frameworks may result in greater structural deformation under functional loading, potentially influencing stress transmission at the implant–bone interface.

Although finite element analysis (FEA) has been widely used to evaluate biomechanical behavior in implant-supported prostheses (21–23), most investigations have focused on metallic frameworks or uniform implant diameters. Limited evidence exists regarding the combined influence of PEEK framework behavior, anterior implant diameter variation, and distal cantilever configuration—clinical conditions frequently encountered in cases with reduced anterior bone availability (24–26).

Therefore, the purpose of this study was to quantitatively evaluate the influence of distal cantilever extension and anterior implant diameter (3.5 mm vs. 4.5 mm) on framework von Mises stress and peri-implant cortical bone response in a PEEK-based mandibular All-on-Four prosthesis under vertical and oblique loading conditions using three-dimensional finite element analysis.

The null hypothesis was that distal cantilever presence and anterior implant diameter (3.5 mm vs. 4.5 mm) would not significantly influence peak framework von Mises stress, crestal cortical bone von Mises stress, or maximum principal cortical strain in a PEEK-based mandibular All-on-Four prosthesis under vertical and oblique loading conditions.

## 2 Materials and methods

### 2.1 Study design

A three-dimensional finite element analysis (FEA) was performed to evaluate the biomechanical behavior of a mandibular All-on-Four prosthesis fabricated from polyetheretherketone (PEEK). The study quantified the influence of distal cantilever presence and anterior implant diameter (3.5 mm vs. 4.5 mm) on framework stress and peri-implant cortical bone response under static loading conditions.

### 2.2 Geometric modeling and implant configuration

**Figure 1:**
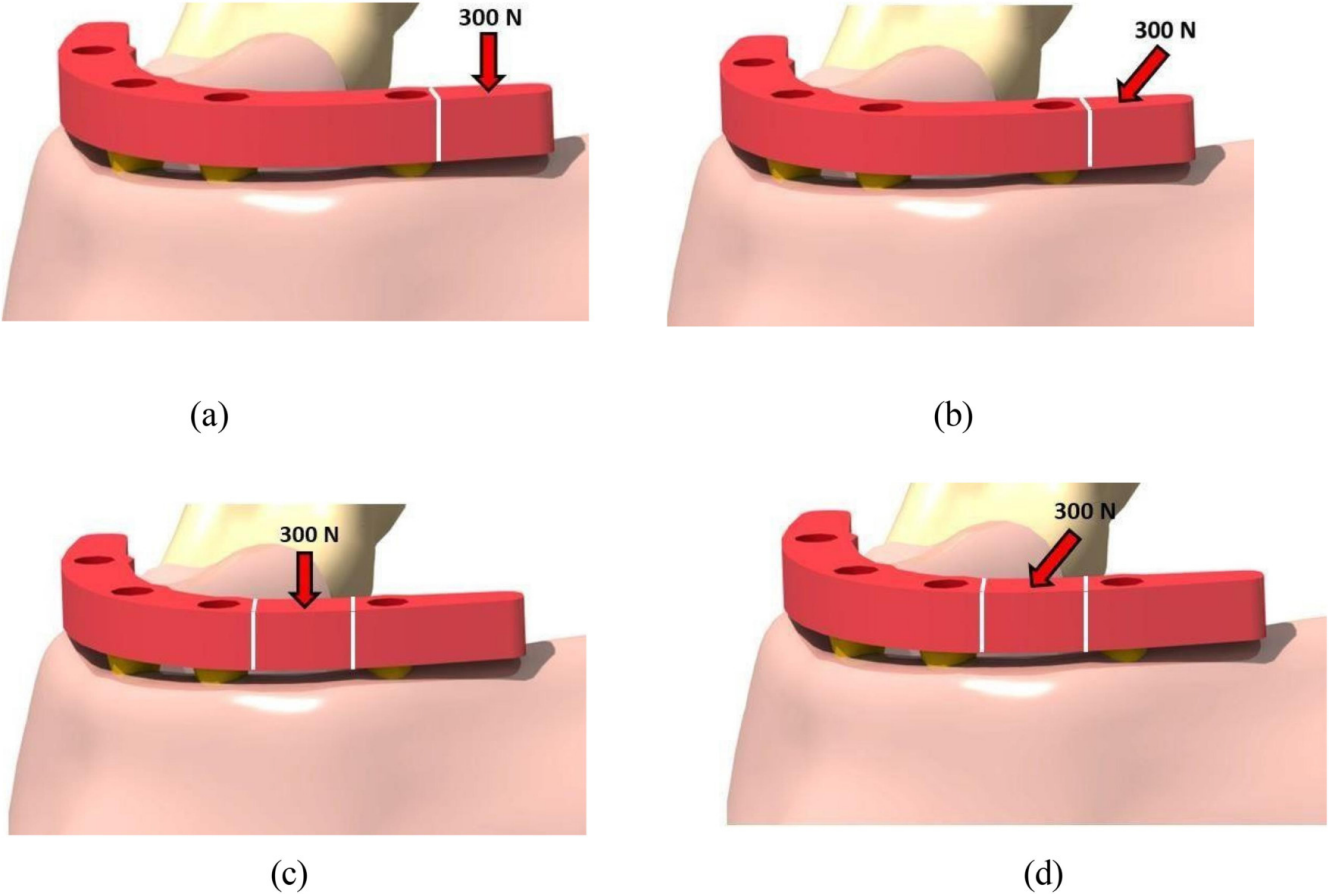
Boundary conditions and load application scheme. The inferior surface of the mandible was fixed in all degrees of freedom. A 300 N load was applied at the premolar and molar regions under vertical (0°) and 30° oblique loading conditions. **(a)** Standard-NC configuration. **(b)** Mixed-NC configuration. **(c)** Standard-C configuration. **(d)** Mixed-C configuration. NC, non-cantilever; C, cantilever.

A three-dimensional edentulous mandibular model was constructed using standardized anatomical dimensions. Cortical and cancellous bone were modeled as separate layers to represent structural heterogeneity. The cortical bone was modeled as a uniform outer shell surrounding the cancellous core (Figure 1).

Four implant-supported configurations were developed:

- **Standard-NC:** 4.5 mm anterior implants, no cantilever
- **Mixed-NC:** 3.5/4.5 mm anterior implants, no cantilever
- **Standard-C:** 4.5 mm anterior implants, with cantilever
- **Mixed-C:** 3.5/4.5 mm anterior implants, with cantilever

In all configurations, two anterior axial implants and two posterior tilted implants were positioned according to the All-on-Four protocol. Cantilever length was standardized in cantilevered models to isolate the effect of implant diameter.

### 2.3 Material properties

All materials were assumed to be homogeneous, isotropic, and linearly elastic (21–23). Elastic modulus and Poisson's ratio values for cortical bone, cancellous bone, titanium implants, and PEEK framework were assigned from previously published literature (11–22) (Table 1).

**Table 1:** Mechanical properties of materials used in the finite element model.

Component	Elastic modulus (GPa)	Poisson's ratio	Tensile strength (MPa)	Compressive strength (MPa)
Cortical bone	14.8	0.30	50–150	130–230
Cancellous bone	0.55	0.30	10–100	2–12
PEEK framework	3.5	0.40	90–100	100–120
Titanium implant/abutment	116	0.34	897–1,170	848–1,070

Material strength values are reported for contextual interpretation and were not used as failure criteria in the linear elastic model.

Material strength values were included for contextual interpretation of stress magnitudes but were not used as failure criteria, as the present analysis did not simulate material plasticity or fatigue.

### 2.4 Contact and boundary conditions

A fully bonded interface was assumed between implants and surrounding bone to simulate complete osseointegration.

The inferior border of the mandible was fixed in all degrees of freedom to prevent rigid body motion. These constraints are consistent with commonly accepted FEA protocols in implant biomechanics (21–23).

### 2.5 Loading conditions

Each model was subjected to a 300 N static load under four loading scenarios:

- 0° (vertical) loading at the premolar region
- 0° (vertical) loading at the molar region
- 30° oblique loading at the premolar region
- 30° oblique loading at the molar region

The oblique load was applied at 30° relative to the vertical axis to simulate non-axial functional forces encountered during mastication.

The analysis was performed under static conditions; dynamic effects, time-dependent material behavior, and cyclic loading were not simulated.

### 2.6 Mesh and convergence strategy

A meshless multi-pass adaptive analysis approach was employed using Sim Solid software (24, 25). Instead of traditional element-based mesh refinement, solution accuracy was achieved through polynomial enrichment in regions of high stress gradients.

Convergence was considered achieved when the variation in peak von Mises stress between successive adaptive passes was less than 5% (Table 2).This threshold was applied uniformly across all models to ensure numerical consistency. Because stress intensification can occur at geometric discontinuities and constraint boundaries in numerical simulations, peak values confined to non-physiologic constraint interfaces were excluded from analysis.

**Table 2:** Adaptive solution passes required for convergence of each implant configuration.

Model	Implant configuration	Adaptive solution passes
Standard-NC	4.5 mm anterior implants, no cantilever	4
Mixed-NC	3.5/4.5 mm anterior implants, no cantilever	4
Standard-C	4.5 mm anterior implants, with cantilever	5
Mixed-C	3.5/4.5 mm anterior implants, with cantilever	5

Convergence was achieved when variation in peak von Mises stress between successive adaptive passes was <5% for all models.

### 2.7 Outcome measures

Primary outcome measures included:

1. Peak von Mises stress within the PEEK framework
2. Peak von Mises stress within the crestal cortical bone
3. Maximum principal strain in peri-implant cortical bone

Von Mises stress was selected to evaluate overall framework material response under combined loading states. For cortical bone, both von Mises stress and maximum principal strain were assessed to characterize mechanical demand. Maximum principal strain was recorded because tensile strain in crestal cortical bone has been associated with peri-implant remodeling and overload in previous biomechanical studies.

Although trabecular bone was included in the model geometry and assigned appropriate material properties, quantitative analysis focused on crestal cortical bone. Peak stress concentration in implant-supported prostheses is consistently reported at the cortical crest adjacent to the implant neck, making this region clinically relevant for early overload assessment.

Global framework displacement was computed but was not considered a primary biomechanical indicator and is therefore not emphasized in the present analysis.

### 2.8 Statistical considerations

As this was a deterministic finite element simulation study, no inferential statistical testing was performed. Comparative evaluation between configurations was conducted using percentage differences in peak stress and strain values.

## 3 Results

Four implant–prosthesis configurations were evaluated under 300 N vertical (0°) and oblique (30°) loading applied at premolar and molar regions. Outcomes included peak von Mises stress in the PEEK framework, peak von Mises stress in the crestal cortical bone, and maximum principal strain in peri-implant cortical bone.

### 3.1 Peak von Mises stress in the PEEK framework

**Figure 2:**
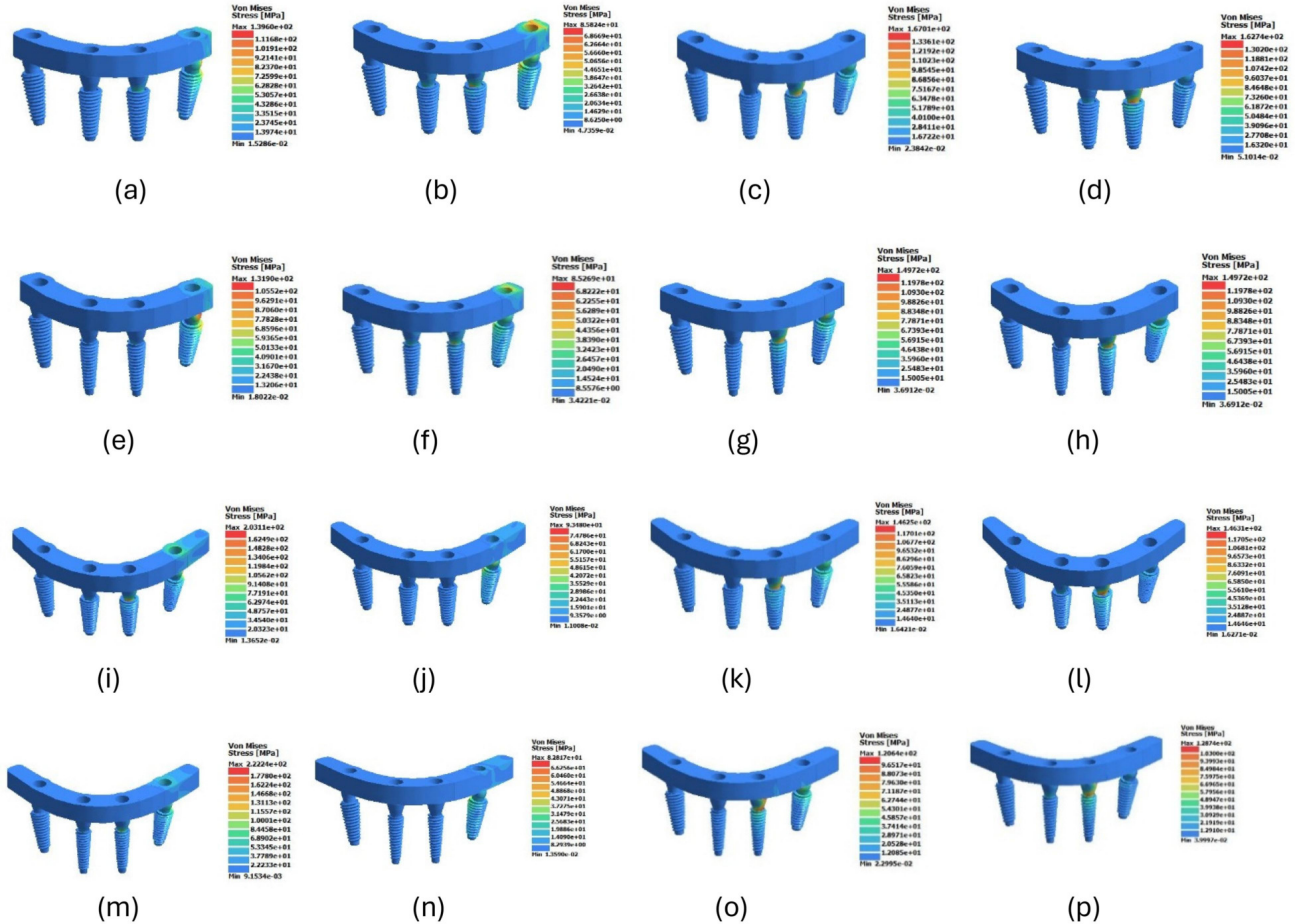
Three-dimensional von Mises stress distribution (MPa) within the PEEK framework under 300 N loading. Subfigures are arranged as follows: **(a–d)** Standard-NC: vertical premolar, oblique premolar, vertical molar, oblique molar. **(e–h)** Mixed-NC: vertical premolar, oblique premolar, vertical molar, oblique molar. **(i–l)** Standard-C: vertical premolar, oblique premolar, vertical molar, oblique molar. **(m–p)** Mixed-C: vertical premolar, oblique premolar, vertical molar, oblique molar. Color gradients represent increasing von Mises stress magnitude (MPa). Warmer colors indicate higher stress concentration within the PEEK framework. Peak stress localization was observed predominantly at the distal implant–abutment interface, particularly in cantilevered configurations under premolar vertical loading.

Peak von Mises stresses recorded in the PEEK framework under vertical (0°) and 30° oblique loading are summarized in Table 3 and Figure 2.

**Table 3:** Peak von Mises stress (MPa) in the PEEK framework under vertical (0°) and 30° oblique loading.

Model	Implant configuration	Premolar vertical (0°)	Premolar oblique (30°)	Molar vertical (0°)	Molar oblique (30°)
Standard-NC	4.5 mm anterior implants, no cantilever	139.60	85.82	167.01	162.74
Mixed-NC	3.5/4.5 mm anterior implants, no cantilever	131.90	85.27	149.72	149.72
Standard-C	4.5 mm anterior implants, with cantilever	203.11	93.48	146.25	146.31
Mixed-C	3.5/4.5 mm anterior implants, with cantilever	222.24	82.82	120.64	128.74

#### 3.1.1 Premolar vertical loading (0°)

The Standard-NC configuration demonstrated a peak stress of 139.60 MPa.

The Mixed-NC model showed a 5.5% reduction (131.90 MPa).

Introduction of a distal cantilever increased framework stress substantially. The Standard-C model exhibited a 45.5% increase (203.11 MPa), while the Mixed-C configuration demonstrated the highest stress (222.24 MPa), corresponding to a 59.2% increase relative to the non-cantilevered baseline.

#### 3.1.2 Premolar oblique loading (30°)

Under oblique loading, the Standard-NC configuration showed a peak stress of 85.82 MPa.

The Mixed-NC model showed negligible change (−0.6%).

The Standard-C configuration demonstrated an 8.9% increase (93.48 MPa), whereas the Mixed-C model showed a 3.5% reduction relative to baseline.

#### 3.1.3 Molar vertical loading (0°)

Under molar vertical loading, the highest stress was observed in the Standard-NC configuration (167.01 MPa).

All other configurations demonstrated lower stress values: Mixed-NC (−10.4%), Standard-C (−12.4%), and Mixed-C (−27.8%).

#### 3.1.4 Molar oblique loading (30°)

Under molar oblique loading, the Standard-NC configuration exhibited 162.74 MPa.

Stress values were reduced in all other configurations: Mixed-NC (−8.0%), Standard-C (−10.1%), and Mixed-C (−20.9%).

### 3.2 Peak von Mises stress in crestal cortical bone

**Figure 3:**
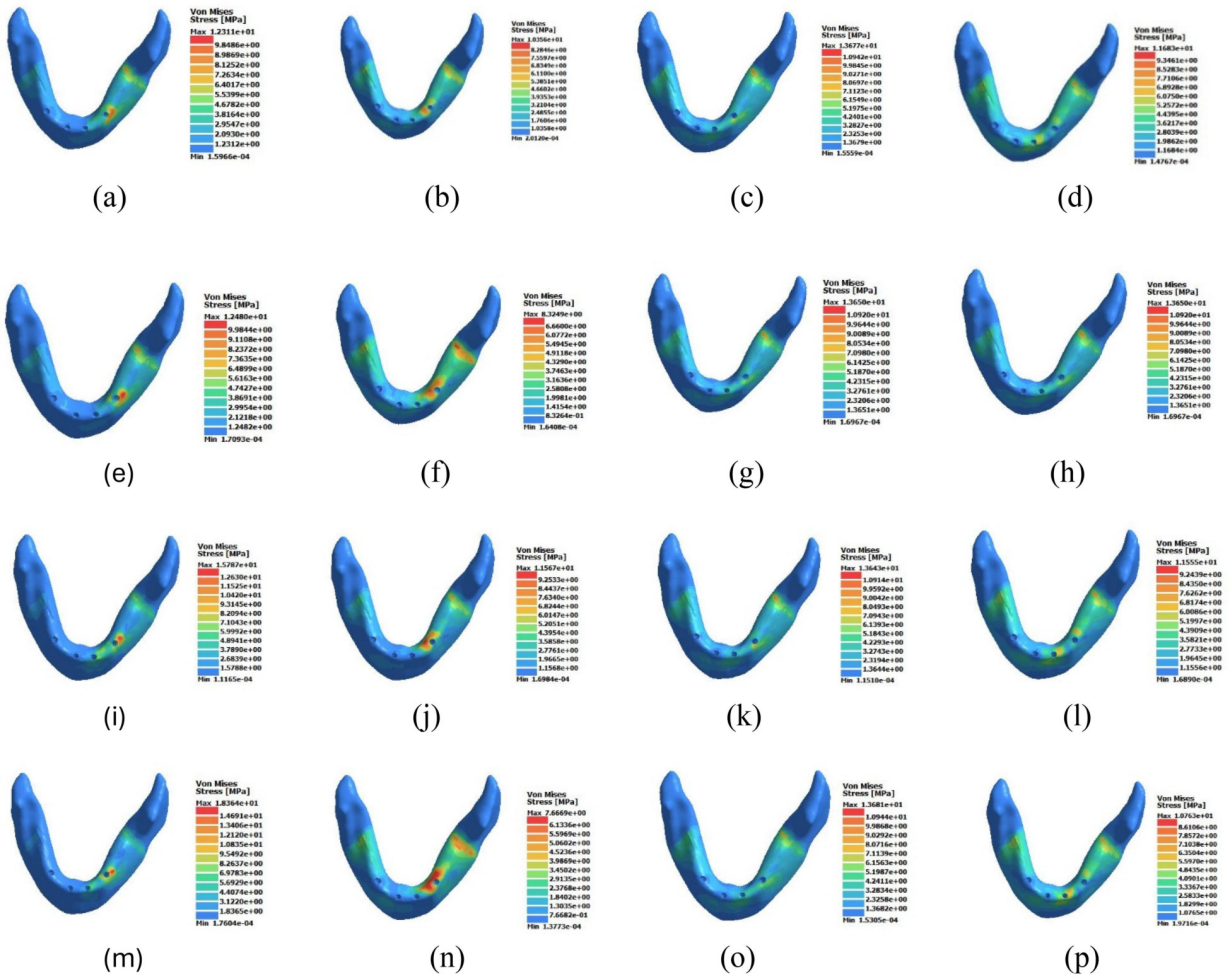
Three-dimensional von Mises stress distribution (MPa) within the crestal cortical bone under 300 N loading. Subfigures are arranged as follows: **(a–d)** Standard-NC: vertical premolar, oblique premolar, vertical molar, oblique molar. **(e–h)** Mixed-NC: vertical premolar, oblique premolar, vertical molar, oblique molar. **(i–l)** Standard-C: vertical premolar, oblique premolar, vertical molar, oblique molar. **(m–p)** Mixed-C: vertical premolar, oblique premolar, vertical molar, oblique molar. Color gradients represent increasing von Mises stress magnitude (MPa). Warmer colors indicate higher stress concentration within the crestal cortical bone surrounding the implant neck region.

Peak von Mises stress values in the crestal cortical bone are presented in Table 4 and Figure 3.

**Table 4:** Peak von Mises stress (MPa) in the crestal cortical bone under vertical (0°) and 30° oblique loading.

Model	Implant configuration	Premolar vertical (0°)	Premolar oblique (30°)	Molar vertical (0°)	Molar oblique (30°)
Standard-NC	4.5 mm anterior implants, no cantilever	12.31	10.36	13.68	11.68
Mixed-NC	3.5/4.5 mm anterior implants, no cantilever	12.48	8.32	13.65	13.65
Standard-C	4.5 mm anterior implants, with cantilever	15.79	11.57	13.64	11.56
Mixed-C	3.5/4.5 mm anterior implants, with cantilever	18.36	7.67	13.68	10.76

#### 3.2.1 Premolar vertical loading (0°)

The Standard-NC model demonstrated a peak cortical stress of 12.31 MPa.

The Mixed-NC configuration showed minimal change (+1.4%).

Cantilever presence resulted in increased cortical stress. The Standard-C configuration exhibited a 28.3% increase (15.79 MPa), and the Mixed-C model showed the highest stress (18.36 MPa), representing a 49.1% increase relative to baseline.

#### 3.2.2 Premolar oblique loading (30°)

Under oblique loading, the Standard-NC model recorded 10.36 MPa.

The Mixed-NC model showed a 19.7% reduction, while the Standard-C configuration exhibited an 11.7% increase.

The Mixed-C model demonstrated a 25.9% reduction relative to baseline.

#### 3.2.3 Molar vertical loading (0°)

Under molar vertical loading, peak cortical stress in the Standard-NC configuration was 13.68 MPa.

Stress values remained nearly unchanged across configurations, with variations less than 1% among all models.

#### 3.2.4 Molar oblique loading (30°)

Under molar oblique loading, the Standard-NC model exhibited 11.68 MPa.

The Mixed-NC configuration demonstrated a 16.9% increase, while Standard-C showed a slight reduction (−1.0%).

The Mixed-C model exhibited a 7.9% reduction relative to baseline.

### 3.3 Maximum principal strain in cortical bone

Maximum principal strain values in the crestal cortical bone under vertical (0°) and 30° oblique loading are presented in Table 5.

**Table 5:** Maximum principal strain in crestal cortical bone under vertical (0°) and 30° oblique loading.

Model	Implant configuration	Premolar vertical	Premolar oblique	Molar vertical	Molar oblique
Standard-NC	4.5 mm anterior, no cantilever	0.0028	0.0025	0.0039	0.0035
Mixed-NC	3.5/4.5 mm anterior, no cantilever	0.0036	0.0032	0.0051	0.0046
Standard-C	4.5 mm anterior, with cantilever	0.0049	0.0044	0.0067	0.0061
Mixed-C	3.5/4.5 mm anterior, with cantilever	0.0061	0.0056	0.0080	0.0073

Values represent peak maximum principal strain (dimensionless) recorded in the crestal cortical bone surrounding the implants for each configuration under vertical (0°) and 30° oblique loading at premolar and molar regions. Strain values correspond to localized maxima within physiologically relevant peri-implant regions.

#### 3.3.1 Premolar vertical loading (0°)

The Standard-NC configuration demonstrated a maximum principal strain of 0.0028.

The Mixed-NC model showed a 28.6% increase (0.0036).

Introduction of a distal cantilever resulted in marked strain amplification. The Standard-C configuration exhibited a 75.0% increase (0.0049), while the Mixed-C model demonstrated the highest strain (0.0061), corresponding to a 117.9% increase relative to the non-cantilevered baseline.

#### 3.3.2 Premolar oblique loading (30°)

Under oblique loading, the Standard-NC configuration recorded a strain of 0.0025.

The Mixed-NC model demonstrated a 28.0% increase (0.0032).

The Standard-C configuration showed a 76.0% increase (0.0044), while the Mixed-C model exhibited the highest strain (0.0056), representing a 124.0% increase compared with the baseline.

#### 3.3.3 Molar vertical loading (0°)

Under molar vertical loading, the Standard-NC model demonstrated a strain of 0.0039.

The Mixed-NC configuration showed a 30.8% increase (0.0051).

The Standard-C configuration exhibited a 71.8% increase (0.0067), and the Mixed-C model demonstrated the highest strain (0.0080), corresponding to a 105.1% increase relative to Standard-NC.

#### 3.3.4 Molar oblique loading (30°)

Under molar oblique loading, the Standard-NC configuration recorded a strain of 0.0035.

The Mixed-NC model showed a 31.4% increase (0.0046).

The Standard-C configuration exhibited a 74.3% increase (0.0061), while the Mixed-C model demonstrated the highest strain (0.0073), representing a 108.6% increase relative to baseline.

### 3.4 Displacement

Maximum framework displacement was greatest in cantilevered configurations and followed trends similar to stress amplification patterns. As displacement does not directly represent material stress or bone overload, detailed values are not further emphasized.

## 4 Discussion

The present finite element analysis evaluated the biomechanical behavior of PEEK frameworks in All-on-4 configurations with varying anterior implant diameters and distal cantilever extension. The null hypothesis was rejected, as both cantilever presence and mixed anterior implant diameter significantly influenced stress magnitude within the prosthetic framework and surrounding cortical bone.

### 4.1 Influence of distal cantilever extension

Distal cantilever extension produced the most pronounced increase in framework stress, with peak von Mises values rising by up to 59% under premolar vertical loading (222.24 MPa vs. 139.60 MPa). This finding is consistent with fundamental biomechanical principles, as cantilever arms amplify bending moments at the distal implant interface. The increase was also reflected in cortical bone stress and principal strain values, indicating that cantilever geometry has a dominant influence on load transmission patterns.

Importantly, this effect was more pronounced under vertical loading than oblique loading in several configurations, suggesting that bending moment amplification rather than shear dominance governs stress escalation in cantilevered models.

The observed localization of stress at the distal implant–abutment interface is consistent with established beam mechanics and prior computational analyses of cantilevered implant prostheses (27–29).

These findings reinforce the mechanical disadvantage introduced by posterior cantilever extension resulting in increased risk of mechanical complications, including screw loosening, framework fracture, and marginal bone loss, which remain primary maintenance concerns (30–32).

### 4.2 Effect of mixed anterior implant diameters

Mixed-diameter anterior implants (3.5/4.5 mm) demonstrated higher framework stresses when combined with cantilever extension. This suggests an interaction effect, where reduced anterior cross-sectional stiffness may magnify distal bending stresses in the presence of a cantilever. However, in non-cantilevered models, mixed diameters produced only modest changes in stress magnitude, indicating that implant diameter alone does not critically affect stress distribution unless combined with distal extension.

Thus, the “compounding effect” is more accurately interpreted as a biomechanical interaction between cantilever length and anterior implant stiffness.

This finding aligns with previous biomechanical studies reporting that implant diameter plays a role in stress distribution, often exerting a greater influence than implant length (33–35). These findings support clinical recommendations favoring the use of wider anterior implants whenever anatomical conditions permit, especially in full-arch rehabilitations subjected to complex loading patterns.

### 4.3 Framework stress in relation to material strength

Peak von Mises stresses within the PEEK framework reached 222.24 MPa in the most unfavorable configuration. These values exceed the reported tensile strength of unfilled PEEK (90–100 MPa) (36). However, finite element peak stresses represent localized stress concentrations within a linear elastic model and may overestimate true material response at geometric discontinuities or mesh-sensitive regions.

The reported tensile strength values are derived from standardized uniaxial testing of bulk specimens and do not directly correspond to complex, multi-axial stress states in implant-supported prostheses. Therefore, the present findings should be interpreted comparatively rather than as direct predictors of clinical fracture. Nevertheless, the substantial increase in peak stress associated with cantilever extension indicates a clear mechanical disadvantage that warrants cautious prosthetic planning when using lower modulus materials.

### 4.4 Cortical bone stress and strain

Maximum cortical bone stress reached 18.36 MPa, which remains well below reported compressive strength values of cortical bone (130–230 MPa). This suggests that under static loading conditions, catastrophic compressive bone failure is unlikely.

However, bone is more sensitive to tensile and shear stresses than to compressive loading. While the present study evaluated von Mises stress as a global indicator, future analyses incorporating maximum principal tensile stress may provide a more clinically relevant assessment of peri-implant bone risk.

Principal strain values increased by over 100% in cantilevered mixed-diameter models under molar vertical loading (0.0080 vs. 0.0039), indicating amplified deformation at the crestal bone. Although these strain magnitudes remain within physiologic remodeling thresholds, their relative increase underscores the biomechanical impact of distal extension.

### 4.5 Influence of elastic modulus

PEEK possesses a substantially lower elastic modulus (3.5 GPa) compared to titanium (116 GPa). Lower modulus materials allow greater elastic deformation, which can redistribute stress over a wider area. However, this same flexibility increases bending under cantilever loading, resulting in higher localized framework stress.

The present study did not include a titanium framework comparison. A direct finite element comparison between PEEK and titanium frameworks would clarify whether the lower modulus of PEEK reduces peak bone stress or merely shifts stress concentration to the framework. Such comparative analysis is recommended for future work.

### 4.6 Methodological considerations

The model employed linear elastic material properties and static loading conditions. Plastic deformation, fatigue behavior, and time-dependent viscoelastic effects of PAEK materials were not simulated. Consequently, the dampening characteristics sometimes attributed to PEEK are not represented in this analysis.

Finite element peak stress values are sensitive to mesh density and geometric transitions. Although convergence was achieved (<5%), localized stress amplification may occur at implant–framework interfaces. Therefore, results should be interpreted in a comparative biomechanical context rather than as absolute failure thresholds.

### 4.7 Clinical implications

Within the limitations of this static linear analysis, minimizing distal cantilever extension appears more critical than modifying anterior implant diameter when using PEEK frameworks. Cantilever elimination consistently reduced framework stress, cortical bone stress, and principal strain values across loading scenarios.

These findings support cautious cantilever use when employing lower modulus framework materials and reinforce established prosthetic design principles in implant-supported full-arch rehabilitation.

### 4.8 Limitations and future directions

Several limitations should be acknowledged:

1. Static linear elastic assumptions were employed. Cyclic loading, fatigue behavior, and time-dependent viscoelastic properties of PEEK were not modeled (37).
2. A fully bonded implant–bone interface was assumed, representing complete osseointegration.
3. Standardized mandibular geometry and homogeneous isotropic material properties were used.
4. Although trabecular bone was included, quantitative analysis focused on cortical bone.
5. Direct comparison with metallic frameworks (e.g., titanium) was not performed.

Future investigations could incorporate:

- Comparative finite element simulations using metallic frameworks,
- Multibody dynamic models simulating full chewing cycles,
- Experimental validation of fatigue performance in PAEK-based systems,
- Separate evaluation of tensile and compressive principal stresses at the bone–implant interface.

## 5 Conclusion

Within the limitations of this three-dimensional finite element analysis, distal cantilever extension was the primary determinant of biomechanical amplification in a PEEK-based mandibular All-on-Four prosthesis. Cantilever presence increased peak framework stress by approximately 46%–59% under premolar vertical loading and increased crestal cortical stress by up to 49% compared with the non-cantilevered baseline configuration. Under posterior vertical loading, maximum principal cortical strain increased by approximately 72%–105%, with the highest strain observed in the cantilevered mixed-diameter model.

Reduction of anterior implant diameter exerted a secondary but additive effect, increasing cortical strain by approximately 28%–31% in non-cantilevered models and further amplifying strain when combined with cantilever extension.

These findings indicate that in low-modulus framework systems such as PEEK, biomechanical response is highly sensitive to cantilever geometry, while implant diameter reduction compounds stress and strain concentration in the peri-implant cortical bone. Careful prosthetic design and cantilever minimization are therefore critical considerations in full-arch rehabilitations utilizing PEEK frameworks.

## Data Availability

The original contributions presented in the study are included in the article/Supplementary Material, further inquiries can be directed to the corresponding author.
